# Clinical Characteristics, Renal Involvement, and Therapeutic Options of Pediatric Patients With Fabry Disease

**DOI:** 10.3389/fped.2022.908657

**Published:** 2022-06-01

**Authors:** Carmen Muntean, Iuliana Magdalena Starcea, Cristina Stoica, Claudia Banescu

**Affiliations:** ^1^Department of Pediatrics I, George Emil Palade University of Medicine, Pharmacy, Science, and Technology of Targu Mures, Targu Mures, Romania; ^2^Department of Pediatric Nephrology, Sf Maria Emergency Hospital for Children Iasi, University of Medicine and Pharmacy Grigore T. Popa Iasi, Iasi, Romania; ^3^Pediatric Nephrology Department, Fundeni Clinical Institute, University of Medicine and Pharmacy Carol Davila Bucharest, Bucharest, Romania; ^4^Center for Advanced Medical and Pharmaceutical Research, George Emil Palade University of Medicine, Pharmacy, Science and Technology of Targu Mures, Targu Mures, Romania

**Keywords:** Fabry, kidney, *GLA* gene, biomarker, therapy

## Abstract

Inherited renal diseases represent 20% of the causes of end-stage renal diseases. Fabry disease, an X-linked lysosomal storage disorder, results from α-galactosidase A deficient or absent activity followed by globotriaosylceramide (Gb3) accumulation and multiorgan involvement. In Fabry disease, kidney involvement starts early, during intrauterine life by the Gb3 deposition. Even if chronic kidney disease (CKD) is discovered later in adult life in Fabry disease patients, a decline in glomerular filtration rate (GFR) can occur during adolescence. The first clinical sign of kidney involvement is represented by albuminuria. So, early and close monitoring of kidneys function is required: albuminuria and proteinuria, urinary albumin-to-creatinine ratio, serum creatinine, or cystatin C to estimate GFR, while urinary sediment with phase-contrast microscopy under polarized light may be useful in those cases where leucocyte α-Gal A activity and *GLA* genotyping are not available. Children with Fabry disease and kidney involvement should receive enzyme replacement therapy and nephroprotective drugs (angiotensin-converting enzyme inhibitors or angiotensin receptor blockers) to prevent or slow the progressive loss of kidney functions. Early diagnosis of Fabry disease is important as enzyme replacement therapy reduces symptoms, improves clinical features and biochemical markers, and the quality of life. More importantly, early treatment could slow or stop progressive organ damage in later life.

## Key Lecture

The wide spectrum of signs and symptoms in Fabry disease represents a challenge in its management nowadays, and these include timely diagnosis and early therapy in children, the necessity for biomarkers that are correlated with the earliest changes in histology. Early enzyme-replacement therapy (ERT) and other therapeutic strategies may impact morbidity and mortality in Fabry disease patients. A delay in diagnosis of Fabry disease influences the quality of life and reduces the life expectancy in nontreated patients.

## Introduction

Fabry disease (FD), also known as Anderson-Fabry disease (OMIM #301500), is a multisystem and heterogenous lysosomal storage disease, with an X-linked inheritance pattern characterized by complete or partial deficiency of the lysosomal alpha-galactosidase A (α-Gal A) enzyme activity. The enzymatic defects result in subsequent accumulation of globotriaosylceramide (Gb3 or GL3) and glycosphingolipids within cellular lysosomes, plasma, and urine causing multiorgan damage with life-threatening manifestations (1).

Fabry disease is a multifaceted condition that begins during intrauterine life. Elleder et al. stated that storage material is already present in the fetal kidney (2), while Vedder et al. only investigated placental storage and speculated on storage in fetal organs (3). Usually, even if the affected infants look normal at birth, the clinical signs of the disease will develop gradually once undigested sphingolipids (SLs) such as globotriaosylceramide and globotriaosylsphingosine (lysoGb3 or lysoGL3) accumulate in the body, as a result of the degree of the enzyme deficiency and the severity of the toxic metabolites storage within organs. The clinical impact relies on the severity of the enzyme insufficiency and the tissues in which toxic nonmetabolized intermediates (such as Gb3) accumulate (4). Fabry disease recognizes two major phenotypes: “classic or early-onset” and a mild or “late-onset” phenotype. The classic forms usually occur in childhood or adolescence. Affected males with the late-onset type have residual α-Gal A activity, correlated with a later-onset cardiac and/or renal disease, and lack the major early-onset classical manifestations (5).

Even if a severe phenotype is more frequent in males vs. females, heterozygous women may also exhibit symptoms of varying severity depending on random inactivation of one of the two X chromosomes. The result of a random X chromosome inactivation is represented by a mosaic of cell populations, leading to variable phenotypes from asymptomatic to severely symptomatic heterozygous females (6).

Fabry disease patients require life-long follow-up to detect changes in signs and symptoms. It is characterized by progressive neurological, renal, cardiac, ocular, and dermatological manifestations (7). In Fabry classical disease, kidney involvement starts early, during intrauterine life by the Gb3 deposition. Even if chronic kidney disease (CKD) is discovered later in adult life in Fabry disease patients, a decline in glomerular filtration rate (GFR) can occur during adolescence. So, early and close monitoring of kidney and other organ functions is required. Early diagnosis of Fabry disease is important as enzyme replacement therapy reduces symptoms, and improves clinical features, biochemical markers, and the quality of life. More importantly, early treatment could slow or stop progressive organ damage in later life. Pulmonary involvement is usually mild and expressed by fatigue, persistent cough, obstructive lung disease, and impaired pulmonary function tests (8).

## Epidemiology

Fabry disease is a rare disorder with an estimated overall incidence varying from 1:17000 to 1:117000. The classic form of Fabry disease is estimated to have a prevalence of 1:22000 to 1:40000 in males, while the prevalence for atypical presentation is evaluated to be 1:1000 to 1:3000 in males and 1:6000 to 1:40000 in females (9). A recent meta-analysis of dialysis patients with FD revealed that the classic forms are more frequent than true late-onset forms if pathogenic GLA mutation is considered (10). Also, Choi et al. found a four times higher incidence for classical vs. late-onset Fabry disease (11).

Fabry disease prevalence in different geographical areas is presented in Table 1.

**Table 1:** Prevalence of Fabry disease according to region.

**Region**	**Evaluated period**	**No of the patients screened positive**	**Detection rate/prevalence**	**No of the subjects tested**	**Reference**
Northwestern Italy	July 1, 2003 to June 30, 2005	12	1/ 3,100 late-onset (without novel mutation 1/4.600 males); 1/3,7000 classic phenotype	37,104 boys	(5)
Hungary, Szeged	NL	8 (3 c.427 G>A (p.A143T) +5 intronic sequence change c.-10C < T)	1/5003 Low α-Gal A in 224, retesting 34	40,024 (boys and girls)	(12)
Spain; Galicia	2008	37 genetic variants 1 case p.M290I (c.870G>A)	1/7,575	14,600	(13)
Netherlands	1970–1996	27	1/47,6190 (0.21/100,000) (live births) 1/238,095 (0.42/100,000) (male live births)	12,634,905 No. of live births (6,495,078 No. of male live births)	(14)
North of Portugal	1982–2001	1	1/833,000 live births	NL	(15)
Austria	January 2010 to July 2010	9	1/3,859	34,736 (boys and girls)	(16)
Missouri	January 1 to July 10, 2013	15	1/2,913	43,701 (boys and girls)	(17)
Japan	NL	339	1.25/100,000	NL	(18)
Japan (Fukuoka City and its vicinity)	April 2007 to April 2010	NL	1/7,057 14.17/100.000	21,170 (boys and girls)	(19)
Taiwan	July 2006 to June 2008	75 GLA mutations (73 boys+2 girls); 86% c.936+919G>A	1/1,250 males GLA mutations; 1/1.460 males of the IVS4+919G>A splicing mutation	171,977; (90,288 males; 81,689 females) (86% had the late-onset phenotype: 1 in 1.390 males)	(20)
Australia	1980–1996	36	1/117,000 live births	NL	(16)

## Genetic

*GLA* gene (OMIM 300644) is located on the X chromosome (Xq22.1), consists of eight exons, and encodes for alpha-galactosidase A (enzyme commission number EC 3.2.1.22), a lysosomal enzyme. According to ClinVar, more than 900 variants in the *GLA* gene have been identified so far^1^.

*GLA* gene mutation will lead to the deficiency of the alpha-galactosidase A (α-Gal A) enzyme. The most frequent *GLA* mutations are missense followed by nonsense mutations, but also deletions, duplication, insertions, frameshift, and splice-site mutations are observed (11, 21).

It was suggested that classic forms of the Fabry disease are produced by mutations that lead to complete loss of function of the gene, whereas late-onset disease and milder phenotypes are produced by mutations that result in the amino acid change (11).

Based on different reports *GLA* p.R112C, p.L129P, p.C142W, p.P205L, p.H46R,p, M42V, pD266N, p.G271C, p.G274R, p.S297R, p.D322E, p.W349R, p.W226^*^, p.R220^*^, p.R227^*^, p.K240Efs^*^8, p.S345Rfs^*^28, p.T412Sfs^*^37. p.G266Vfs^*^8, p.L268fs^*^1, p.Q99fs^*^23, p.D61Efs^*^32 and p.L344fs ^*^31 are considered classical variants; while p.M296I, p.R301Q, p.R112H, p.N215S, F113L, I91T, p.R363H, and L310V are considered late-onset variants (11, 22–28). GLA p.R112H and p.M296I are considered as variants of uncertain significance (29).

Some *GLA* variants may cause amino acid substitutions and/or low α-Gal A enzyme activity and it is still debated whether these mutations may cause Fabry symptoms and are considered variants of unknown significance at the moment for example p.E66Q (30, 31), p.D313Y (32–35), p.S126G (36), p.R118C (37, 38), and p.A143T (39, 40).

Detection of a known *GLA* pathogenic variant by sequencing analysis allows for a definitive Fabry disease diagnosis. If sequence analysis identifies a novel variant or variants of unknown significance a comprehensive examination is necessary and may include clinical symptoms, the course of disease, family history, α-Gal A enzyme activity level, lysoGb3 level in the blood, and renal biopsy in cases with additional symptoms compatible with Fabry disease (27) considering that not all result in Fabry disease, as many of them are benign ones or polymorphisms without clinical implications (41). Also, there is a wide phenotypic spectrum, even among family members confirmed with a similar genetic mutation (42) therefore a rigorous screening should be done.

Fabry disease has an X-linked pattern of inheritance, usually, the *GLA* mutation is transmitted to the boys through a heterozygous mother. A heterozygous female for *GLA* gene mutation may have affected boys (50%) and healthy boys (50%), and each daughter has a 50% chance of being a heterozygote. An affected father will not transmit the disorder to his son. The daughters of an affected father with Fabry disease will be heterozygotes. Negative family history of Fabry disease does not rule out the diagnosis.

## Physiopathology

The lysosomal hydrolase alpha-galactosidase A (α-Gal A) deficiency will lead to the systemic progressive lysosomal accumulation of complex glycosphingolipids with terminal α-galactosyl moieties, mainly globotriaosylceramide (Gb3) and its deacylated, amphiphilic metabolite, namely globotriaosylsphingosine (lysoGb3), and to a lesser extent, galactosylceramide and other derivatives (43).

Substrate accumulation within lysosomes in the cells of different tissues promotes various pathogenic mechanisms in which are implicated different mediators leading to multisystem lesions, resulting in clinical manifestations of the disease as well as the development of complications that reduce the quality of life (44).

Accumulation of Gb3 results in characteristic lysosomal deposits (Fabry inclusions) in different organs and cell types, known as myelin figures and zebra bodies (45) leading to cell death, with progression to fibrosis and irreversible organic damage and reducing the average life expectancy by 10 years at women and by 25 years at men (41).

It has been suggested that lysoGb3 may represent an important pathogenic factor in Fabry disease. LysoGb3 increases the proliferation of smooth muscle cells leading to increased intima-media thickness and arterial stiffness in patients with Fabry disease (44). In addition, the accumulation of lysoGb3 will deteriorate nociceptive neurons manifested as neuropathic pain (acroparasthesias), and intolerance to heat. Storage of Gb3 and glycosphingolipids in the peripheral nervous system causes dysregulation of vascular tone, tinnitus, and hearing loss (8). Small fiber neuropathy (SFN), a characteristic of FD, involves small myelinated and unmyelinated neurons. Thinly, myelinated Aδ fibers are particularly affected (45, 46). These Aδ fibers involve a cold sensation and mechanical pain sensitivity to pinprick stimuli. Later, thermal stimuli involve warmth perception and pain sensitivity to heat (C-fibers) (45). Aδ fibers are more susceptible to Gb3-induced damage. The SFN in Fabry disease is an age-dependent and disease severity-dependent neuropathy as well as gender-dependent, males being more severely affected (45). Fabry SFN is diagnosed with impaired cold sensation, neuropathic pain, and intraepidermal nerve fiber density (IENFD) < 5^th^ percentile (45). Higher pain incidence was reported in male patients (60–80%) than in females (41–65%) (47, 48). Early therapy is important to reduce small nerve fiber damage, as they occur in the early stages of the disease. Enzyme replacement therapy (ERT) has been shown to reduce the overall pain scores and neuropathic pain in patients (49). Moreover, the accumulation of lysoGb3 contributes to the early damage of podocytes and fibrosis produced in epithelial cells (43). Deposition of lysoGb3 in podocytes (that primarily are loaded with glycolipids) will increase the expression of cytokine TGF-β1 which enhances the synthesis of an extracellular matrix, inhibiting matrix degradation, and altering cell-cell interaction. TGF β will lead to tissue fibrosis, TGF β being one of the main contributors to the development of renal fibrosis (50). TGF β1, one isoform of TGF β is a profibrotic mediator in various kidney diseases and causes both tubular and glomerular epithelial cell-to-mesenchymal transition. TGF-β signaling interacts with other signaling pathways (for example TGF-β/Smad) for mediating the fibrotic process (51). The function of podocytes is influenced by inflammatory cytokines level, which is influenced also by lysoGb3. The damage of podocytes will be followed by podocyte loss and glomerulus resulting in CKD with proteinuria and glomerulosclerosis. Chien et al. reported that accumulation of Gb3 in the kidney starts early during fetal development, as demonstrated by elevated urinary Gb3 levels at birth in male newborns with Fabry disease (52).

Also, renal tubular cells, glomerular endothelial, mesangial, and interstitial cells are affected. Furthermore, increased levels of lysoGb3 inhibit the endothelial nitric oxide synthase (eNOS) and thereby may be involved in the dysfunction of the endothelium (50, 53). Accumulation of Gb3 produces dysregulation of the enzyme nitric oxide endothelial synthase (eNOS) leading to the formation of oxidant species derived from nitric oxide, which may result in vasculopathy (50, 53). Storage of Gb3 and lysoGb3, within lysosomes, also involves cardiac cells (for example cardiomyocytes, valvular fibroblasts) (54). Increased levels of lysoGb3, will lead to cardiomyocyte hypertrophy and ischemia (54) causing a higher risk for cerebrovascular accidents and coronary microvascular disease; cardiac damage: dysrhythmias (tahy-/bradycardia), EKG abnormalities, hypertrophic cardiomyopathy, and valvular dysfunction (8).

## Clinical Features and Renal Symptoms in Children With Fabry Disease

The clinical symptoms of Fabry disease may present at any age, in children and adults (55, 56).

According to the residual GLA enzyme activity of normal value, it may be graded as residual (1–5% of normal values) or no residual (<1% of normal values), or nearly complete deficiency of α-Gal A activity (57). There are two different types of Fabry disease, the early-onset type and the late-onset type. Usually, the early-onset type occurs mostly in males with absent or nearly complete deficiency of α-Gal A activity, while the late-onset type occurs mainly in cases with residual α-Gal A activity (58). The occurrence of early symptoms during childhood is linked to the severity of α-Gal A deficiency. The early-onset type associated with classical phenotype for Fabry disease in male patients involves no residual α-Gal A enzyme activity and begins during childhood.

Clinical symptoms which appear in childhood are represented by gastrointestinal symptoms, neuropathic pain (pain attacks, chronic pain), acroparesthesia, angiokeratoma, hypohidrosis, and corneal opacities (cornea verticillata). Gastrointestinal and eye involvement was reported within the first decade of life. Also, the early median age at onset was observed in males vs. females (at least 2–5 years later in girls vs. boys) (59, 60). Cardiac, renal, and skin manifestations of Fabry disease occurred in the second decade of life (adolescence) (47, 59). Laney et al. (60) and Hopkin et al. (17) reported the mentioned signs and symptoms and their onset early in life (during the toddlerhood and early childhood period).

The main signs and symptoms of Fabry disease observed during childhood are pain (neuropathic pain most frequently localized in palms, soles, and fingertips or acroparesthesia that begin in early childhood) (60, 61), fatigue, gastrointestinal problems (most commonly abdominal pain and diarrhea) (60), reduced or absent sweating (hypohidrosis or anhidrosis), heat or cold or exercise intolerance and angiokeratoma that appears in children and young adolescents (43, 47, 60, 62, 63).

Some studies reported acroparesthesia as the most common initial symptom of Fabry disease with an early age of onset, often not recognized and therefore with a delaying diagnosis (8).

Other symptoms observed during childhood or adolescence may be the corneal sign (presence of corneal whorls or cornea verticillata observed upon slit-lamp examination (64, 65), hearing problems such as hearing loss, tinnitus (66), renal sign (pathological albuminuria or proteinuria, hyperfiltration), cardiac sign (valvular dysfunction, arrhythmias, conduction abnormalities). Also, it was reported delayed growth and it was noticed that the onset of puberty in boys may be affected (60). The signs and symptoms of Fabry disease in children stratified by age are presented in Table 2. The overall quality of life (QoL) of children with Fabry is often considerably reduced and characterized by anxiety, depression, and school absences. The signs and symptoms presented above should alert the pediatricians to the possibility of Fabry disease (62). FD patients require a multidisciplinary approach and organ-specific treatment since the most severe clinical effect is observed in the kidneys, heart, and central nervous system.

**Table 2:** Signs and symptoms of Fabry disease, stratified by age.

**Signs and symptoms**	**Early childhood** **(63)**	**Childhood** **(6, 67)**	**Adolescence** **(6)**	**Age of onset** **(47, 60, 64)**
Kidney
Proteinuria	+/-	+/-	+/-	13.8 years boys, 14.1 years girls (60)
Albuminuria	-	-	+	16.5 years boys, 15.9 years girls (60)
Low FGR (Podocyturia)	+/-	+	+	
Eyes
Cornea verticillata (corneal whorls/retinal vascular tortuosity)	+/-	+	+	8.1 years (64)
Skin and membranes
Decreased sweating (Hypo-/anhidrosis)	+/-	+	+	2.5 years (60)
Angiokeratoma	-	+/-	+	7 years boys, 9.5 years girls (47)
Gastrointestinal system
Gastrointestinal symptoms	+	+	+	1–4.1 years (60)
Heart
Left ventricular hypertrophy	+/-	+/-	+/-	
Arrhythmias and conduction abnormalities	+/-	+/-	+/-	9.3 years boys and 10.3 years boys, 16.9 years girls (60)
Heart valve disease	+/-	-	+/-	8.6 years boys, 14.4 years girls (60)
Nervous system
Limb pain/Acroparesthesias	+	+	+	2–4 years (60)
Episodic pain crises (“Fabry crises”)	+/-	+	+	
Heat or cold intolerance	+/-	+	+	3.5 years (60)
Hearing problems	-	-	+	4 years (60)

A recent paper reported 51 Romanian Fabry patients aged between 11 and 80 years (4). In this Romanian cohort, even if the onset was at about 13 years a significant delay in diagnosis was noticed after the appearance of irreversible damages. Only in two cases from affected families, the diagnosis was established in adolescence, before the onset of complications (4). In their study, Choi et al. reported an incidence of 94 FD cases (10 children-pediatric males) in 50 million people in South Korea, with a four times higher incidence of classical type Fabry disease compared with late-onset. They concluded that Fabry disease, especially the mild, late-onset type is underdiagnosed (11). The period between the mean age at onset and mean age at diagnosis was 1.5 years in the boys. Acroparesthesia was encountered in all children, starting with the age of five in the youngest child (11).

## Renal Involvement

Kidney involvement in Fabry disease is common, its prevalence being 55%, according to Waldek et al. (68). The main cause of death in Fabry patients is cardiovascular disease, followed by cerebrovascular manifestations, with kidney failure or end-stage renal disease (ESRD) being the third most common cause of mortality. Furthermore, chronic kidney disease (CKD) is the main cause of kidney failure in untreated patients with the late-onset or classical type of Fabry (6). The earliest sign of Fabry nephropathy is proteinuria, which is the most significant existing biomarker for progressive decline in GFR (68). Patients with CKD without a clear cause of nephropathy plus concomitant symptoms or signs compatible with FD like neuropathic pain, gastrointestinal problems, angiokeratoma, etc, should trigger testing for Fabry disease.

Subclinical kidney damage appears during childhood and is associated with pathological albuminuria. CKD frequently affects adolescents and adults and is associated with a progressive decline of GFR (6). Increased levels of albuminuria and proteinuria, and a worsening in GFR indicate permanent nephron destruction, in glomerular as well as tubular regions, where interstitial fibrosis, glomerular and arteriolar sclerosis, besides tubular atrophy results in kidney injury (6).

A recent study raised the utility of abdominal ultrasound in patients with CKD of unknown etiology and a positive family history of kidney disease. The presence of parapelvic cysts on ultrasound in these patients can highlight suspicion of Fabry disease, especially since these cysts appear earlier than in the general population (69, 70). Also, Pisani et al. observed a high incidence of parapelvic cysts in Fabry disease patients (up to 43%). Therefore, in patients with a kidney disorder, CKD, or proteinuria of unknown etiology associated with other stigmata of the disease, the diagnosis of Fabry should be considered (57). Other ultrasound abnormalities, namely enlarged kidneys, and increased parenchymal echogenicity have been reported in these patients (71).

It was shown that Gb3 and lysoGb3 are progressively accumulated in different renal cells and to a large extent in podocytes and their quantity increases over time and leads to increased podocyte foot process width and urinary protein excretion, both consistent with morphological and functional podocyte alterations (67). Mauer et al. highlighted that mosaicism of podocytes (with or without Gb3 inclusions) in Fabry disease females is linked to podocyte injury. So, data about podocytes mosaicism are important as they may be useful to identify these patients with increased risk of progressive Fabry nephropathy development (72).

A recent study included 55 males (mean age 27) with classic Fabry disease from which 18 cases were under the age of 18 and revealed that Fabry disease has followed the podocyte injury (67). Previously, the same authors showed that in young patients (under the age of 19) with the classic type of Fabry disease the podocyte Gb3 volume proportion increases (73).

**Figure 1:**
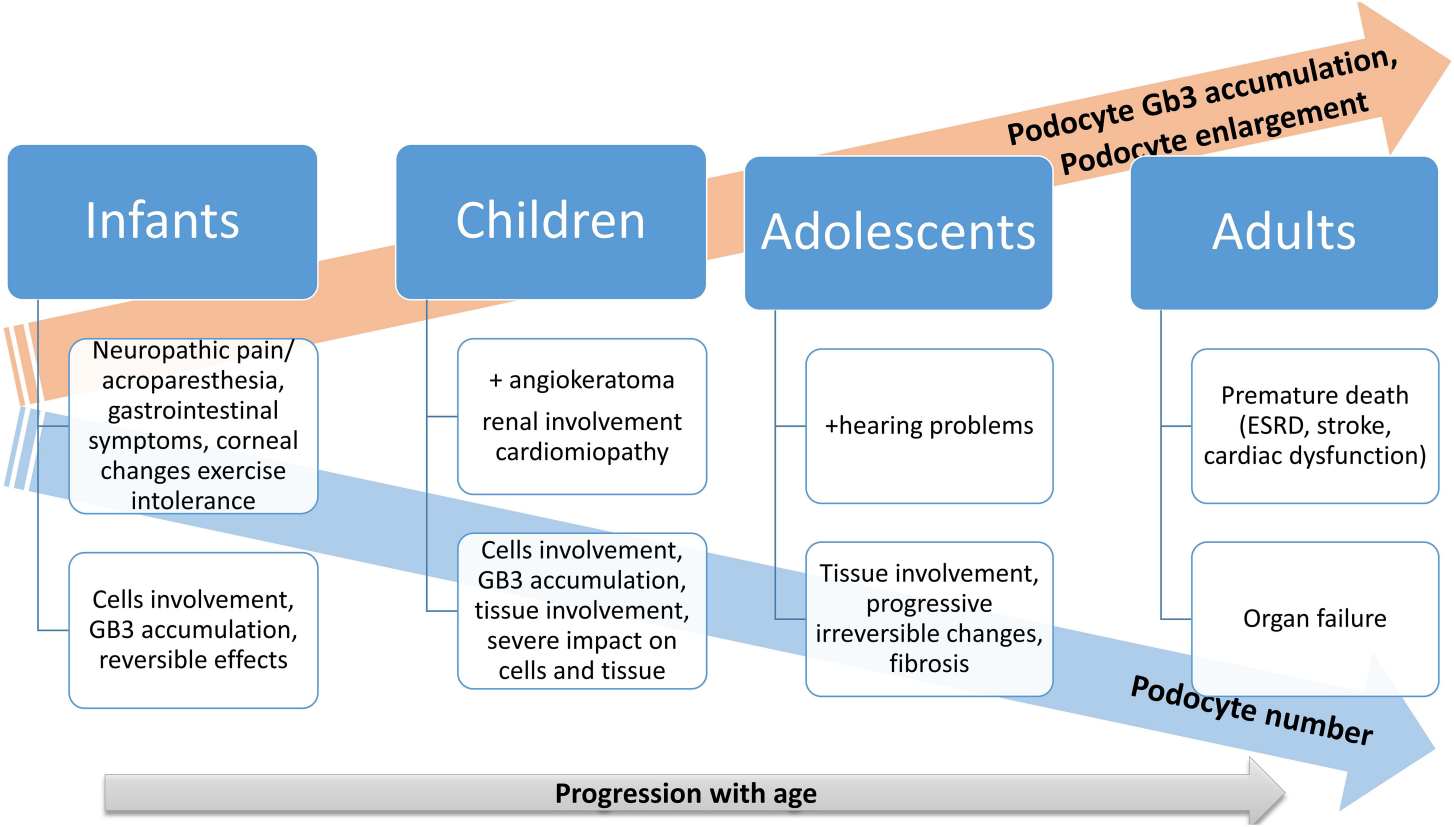
Progression of Fabry disease: a schematic illustration.

There is a physiological podocyte loss with aging, but this loss is more expressed in Fabry disease patients. In the beginning, the podocyte Gb3 accumulation rate is not compensated sufficiently by podocyte enlargement, causing an increasing podocyte Gb3 volume fraction in the first three decades of life. After this, Gb3 continues to accumulate concomitant with podocyte enlargement, along with podocyte loss (67). The same study emphasizes that the podocyte loss process starts quite early in Fabry disease, highlighting that early initiation of enzyme replacement therapy (ERT) is essential, especially before significant proteinuria occurrence (67). The progression of Fabry disease is concomitant with metabolite accumulation resulting in tissue involvement and progressive organs dysfunction as are depicted in Figure 1.

Also, podocyturia should be assessed in Fabry disease cases without renal involvement so far, as podocyturia precedes and is in direct relation to the magnitude of proteinuria (74). The same study suggested that podocyturia in children with Fabry disease may serve as an early biomarker of renal injury (74). In addition, it was reported that podocyturia correlates with the nephropathy's severity in Fabry disease (75–77).

“Podocyturia” evaluation involves an indirect immunofluorescence technique and it is performed only in specialized laboratories. Using special podocyte markers, positive synaptopodin is observed in podocytes in the urine sediment from FD patients (74).

A recent study found that glomerular filtration rate (GFR) and gender are not significant risk factors for pathological proteinuria occurrence, while age over 10 years and podocyturia are the two main risk factors associated with this (74).

Choi et al. investigated Fabry cases from South Korea and observed that all FD pediatric patients presented no proteinuria (94–106 mg/m^2^/day) and normal serum creatinine levels. Three pediatric male patients underwent kidney biopsy before ERT. In one case global sclerosis was observed, as seen on light microscopy (LM). The accumulation of Gb3 was observed in two cases in the mesangial cells on electron microscopy (11). All pediatric patients received ERT. The mean age at ERT initiation was 13.8 years old for boys, with a 3.4-year delay in ERT therapy after the onset of symptoms (11).

Fabry disease in Romanian children is rare and certainly underdiagnosed, compared to the worldwide level. Our Fabry pediatric patients with their main characteristics are presented in Table 3. For eGFR assessment updated Schwartz formula, eGFR=K x Height (cm)/Serum creatinine (mg/dl) k=0,413, was used (78). Within our cohort, one case was diagnosed in the ESRD stage in early life (11 years). A kidney biopsy was only performed in three (43%) cases, and a characteristic FD biopsy pattern was observed. In accordance with literature data, also in our cases, kidney involvement was proved by kidney biopsy before proteinuria occurrence or GFR decline (1 from three biopsies). In our female case, the first symptoms (acroparesthesia) appeared around the age of 11 years, followed by heat intolerance and abdominal pain at the age of 12 and 13, respectively. Non-opioid pain treatment was used only in one case and included most frequently acetaminophen but also neuromodulating anticonvulsant drugs such as gabapentin, as they proved to be efficient for neuropathic and central pain. Quality of life was evaluated in all patients with FD aged 8–17 with the Short Form 36 (SF-36) health-related quality of life survey and brief pain inventory form. ERT was proposed in four cases, but only three accepted it.

**Table 3:** Clinical characteristics and laboratory tests of the affected patients/individuals.

**Patient**	**1**	**2**	**3**	**4**	**5**	**6**	**7**
Age at diagnosis	9.2 years	1.10 years	11 years	17 years	16 years	8 years	14 years
Age at kidney involvement	9 years	no	11 years	16 years	no	no	no
Sex	M	M	M	M	M	M	F
α-gal A activity (N>2.8 μmol/l/h)	0 μmol/l/h	0.1 μmol/l/h	0.25 mol/h/ml	0.17 μmol/l/h	0.91 μmol/l/h	1.68 μmol/l/h	2.2 μmol/l/h
Plasma lysoGb3 (normal range: 0.0–3.5 ng/ml)	101.1 ng/ml	-	124.5 nmol/l = 49.8 ng/ml	26 nmol/l = 10.4 ng/ml	11.24 nmol/l = 4.5 ng/ml	18.7 nmol/l = 5.88 ng/ml	2.5 ng/ml
Genetic testing GLA variants	c.797A>C (p.Asp266Ala)	c.295C>T (p.Gln99Ter)	c.317T>G (p.Leu106Arg)	c.779G>A (p.Gly260Glu)	c.334C>T (p.Arg112Cys)	c.796G>A (p.Asp266Asn)	c.644A>G (p.Asn215Ser)
Protein change	D266A	Q99*	L106R	G260E	R112C	D266N	N215S
Molecular consequence	Missense	Nonsense	Missense	Missense	Missense	Missense	Missense
Affected sibling	Mother, sister, maternal aunt, 3 cousins (2 M, 1 F), Maternal aunt and 2 deceased cousins with severe cardiac + renal illness (dialysis)	Mother and maternal grandfather	Mother	Mother	no	no	Father, Paternal grandmother, parental uncle; 3 parental grandmother sisters
Family history of stroke, heart failure, arrhythmia, etc	Cardiac illness: two maternal aunts and two maternal cousins	Maternal Grandfather: Cardiac insufficiency, deceased after stroke	no	no	no	no	Father-deceased with heart failure, Parental grandmother- deceased after stroke; Uncle with CKD
Heat/cold intolerance	no	no	Heat intolerance	no	no	no	Heat intolerance
Hypo/anhidrosis	no	no	no	no	no	no	Hypohidrosis
Gastrointestinal	no	no	Nausea, epigastralgia	no	no	no	Abdominal pain
Daily neuropathic pain and tingling in hands and feet, acroparesthesia	Burn-like pain in the upper and lower limbs, mostly in the fingers of the lower limbs	Lower limbs paresthesia	Severe burn-like pains in hands and feet	Burn-like pain in hands	no	no	Burn-like pain in hands, acroparesthesia
Depression/anxiety	no	no	depression	no	no	no	Mild depression
Hearing loss	no	no	no	no	no	no	no
Skin	no	no	no	AK (palms, anterior trunk)	AK (umbilicus, upper limbs, posterior trunk)	no	no
Eye	no	Corneal opacities, hypermetropia	Cornea verticillata	Cornea verticillata	no	no	no
Cardiac involvement	mild LVH	no	Subendocardial ischemia, malignant HTN	no	no	no	no
Others	Diabetes mellitus Type 1	no	Acute pulmonary edema	no	no	no	Urinary incontinence
Heart ultrasound	Mild LVH, mild mitral regurgitation	N	Mitral, tricuspid, aortic regurgitation. Mild left ventricular and atrial dilation	N	N	N	N
ECG	N	N	ST depression, T wave inversion	Repolarization anomalies	N	N	N
Abdominal ultrasound	N	N	Atrophic kidneys; Hyperechogenic renal parenchyma, hyperechogenic nodules	Slightly atrophic kidneys; Hyperechogenic renal parenchyma	N	N	N
Proteinuria	no	no	1.2 g/24 h	100 mg/dl	no	no	no
UPCR	N	N	6mg/g	NP	N	N	N
GFR (ml/min/1.73 m^2^)	75.187	85.93	4, PD, HD, followed by kidney transplant	19, kidney transplant	118	130	115.5
Kidney biopsy	glomeruli with increased volume, mesangial proliferation. Significant podocytes damages. Gb3 within glomeruli, podocytes, endothelium	NP	Global sclerosis Lysosomes within glomeruli, endothelium, and podocytes	glomeruli with increased volume and mesangial proliferation, lipid inclusions within glomeruli, tubular cells, smooth muscle cells	NP	NP	NP
Concurrent medications	no	no	Enalaril,Amlodipine, Metoprolol, Prazosin	Enalapril	no	no	Gabapentin Acetaminophen
ERT	Agalsidase beta	no	Agalsidase beta	Agalsidase beta	no	no	Agalsidase beta

M, male; F, female; AK, angiokeratoma; GFR, glomerular filtration rate; UPCR, urine protein/creatinine ratio; ERT, enzyme replacement therapy; N, normal; NP, not performed; HCM, hypertrophic cardiomyopathy; HD, hemodialysis; HTN hypertension; Lyso-Gb3, Globotriaosylsphingosine; LVS, left ventricular hypertrophy; CKD, chronic kidney disease; PD, peritoneal dialysis. *Translation stop codon.

Different studies that included children and adolescents diagnosed with Fabry disease are listed in Table 4.

**Table 4:** Renal involvement in children and adolescents with Fabry disease in different studies.

**Study**	**Geographical area**	**Fabry children included in the study (no)**	**Mean age at diagnosis years (range)**	**Sex** **Males** **Female**	**Renal involvement n (%)**	**Use of ERT**	**Extrarenal findings**	**Additional remarks**
Hopkin et al. (47)	Fabry Registry patients	352	12 (<1–17 years)	194 M, 158 F	16 (4.5%)	Included before ERT's initiation	NP, GI, angiokeratomas, cardiac manifestations,	3 cases with very low eGFR values, 9 with proteinuria, 4 with microalbuminuria
Laney et al. (60)	Review/Europe, China, Fabry Registry	34	2.8 (0.16–4 years)	23 M, 8 F, 3 NL	2 (5.9%)	NL	acroparesthesia/NP, GI, CV, heat/cold intolerance, cardiac signs, angiokeratoma,	globotriaosylceramide inclusions in renal glomerular cells on biopsy, low GFR
Ramaswami et al. (59)	11 countries in Europe	82	12.9 (0.7–17.9 years)	40 M, 42 F	23 (28%)	NL	acroparaesthesia, NP, GI, CV heat/cold intolerance, anhidrosis hypohidrosis, angiokeratoma,	Urinary sign: proteinuria, albuminuria, hematuria
Najafian et al. (73)	Norway, USA	14	12 (4–19 years)	8 M, 6 F	14 (100%)	0	acroparesthesia, corneal opacity, angiokeratoma	Gb3 inclusions in glomerular cells, normal GFR, absent/ low-grade proteinuria
Ries et al. (79)	Germany, UK, Italy, Sweden	35	12.6 (1–21 years)	15 M, 20 F	5 (14.3%)	NL	acroparesthesia, hypohidrosis, CV, NP, GI, depression	5 cases with proteinuria and / or low creatinine clearance
Tøndel et al. (80)	Norway	16	12.7 (5–18 years)	9 M, 7 F	9 (56.25%)	8	acroparesthesia, typical eye changes, hypohidrosis, GI, angiokeratomas	all renal biopsies (9 cases) with Gb3 inclusions in podocytes and distal tubules; proteinuria and albuminuria (5 cases) hyperfiltration (8 cases)
Shen et al. (81)	China	10	7.7 (0.1–16 years)	9 M, 1 F	1 (10%)	4	Pain, early-onset, stroke, hypohidrosis, angiokeratoma, GI symptoms, LVH, arrhythmia	neurological symptoms in 1 boy at 0.7 years; cardiac symptoms in 1 girl at 3.6 years
Furujo et al. (82)	Japan	2	12 (11–13 years)	2 M	0 (0%)	2	Hypo- /anhidrosis, angiokeratoma, CV	urine sediment Gb3 levels were elevated at baseline
Auray-Blais et al. (83)	Canada	32	2–17 years	15 M, 17 F	2 (6.3%)	8	acroparesthesia, hypohidrosis, pain, heat intolerance, diarrhea	massive excretion of Gb3 in cases with nonsense mutation
Marchesoni et al. (84)	Buenos Aires, Argentina	44	14.6 (7–21 years)	20 M, 24 F	3 (6.8%)	3	NP, CV, abdominal pain	Abnormal brain MRI in 7 cases (5 F), 3 received ERT
Present work	Romanian	7	10.6 (1.10–17)	6 M, 1 F	4 (57.1%)	4	NP followed by GI, heart, skin, and eye symptoms	Dialysis and kidney transplant in 2 cases

*M, male, F, female, MRI, magnetic resonance imaging, NP, Neuropathic pain, CV, cornea verticillata, GI, gastrointestinal symptoms, NL, not listed, GFR, glomerular filtration rate*.

## Diagnosis of Fabry Disease

Diagnosis of index cases of Fabry disease is usually delayed and rarely occurs during childhood, and this is due to the lack of specific symptoms (62, 85). The diagnosis of Fabry disease in a proband should include immediately a clinical examination, but also a biochemical and genetic investigation of the relatives both in males and females gender (8).

For boys with clinical signs and symptoms of Fabry disease, it is recommended to investigate α-Gal A activity, a value of < 1% being highly suggestive of Fabry disease and molecular testing for *GLA* gene mutation is necessary. Identification of a known mutation confirmed the diagnosis of Fabry. If a VUS is identified in the *GLA* gene, analysis of lysoGb3 is useful (1). If Fabry disease is suspected in girls *GLA* gene mutation analysis should be performed. In case of identification of a VUS in girls, lysoGb3 is recommended (1).

In their research, Wang et al. stated that heterozygous Fabry females should not be considered just carriers, as they may be symptomatic with severe organs involvement and risk of premature death similar to male Fabry disease patients (8). Similarly, Fernando et al. demonstrated a significant and severe affection for women with Fabry disease (86).

### α-Gal a Enzyme Activity

The activity of the α-Gal A enzyme may be measured by the liquid chromatography-tandem mass spectrometry (LC-MS) method or other methods from dried blood spots (DBSs), plasma, or serum (25, 87). The α-Gal A enzyme activity in blood is a sensitive analysis used for diagnosing affected males, but it has not been reliable for detecting manifesting heterozygous females which may have normal or only slightly decreased α-GAL A activity. The study of Stiles et al. showed that α-Gal A activity in DBS has high sensitivity (100%), but lower specificity (74%) for Fabry disease in males, as not all males with low α-Gal A activities were confirmed to have Fabry disease and recommended the enzyme analysis as the first-tier testing in males (87).

### Globotriaosylceramide (Gb3) in Peripheral Blood Mononuclear Cells

Detection of Gb3 in peripheral blood mononuclear cells (PBMCs) was proposed as a new tool for diagnosis and therapy monitoring of Fabry cases with classic form (88). Detection of Gb3 from PBMCs may represent a non-invasive, time-saving, and low-cost method (89). It may represent an alternative method in children, in whom invasive kidney biopsies are often refused (88). A reduction of Gb3 in PBMCs in Fabry patients was noticed after long-term enzyme replacement treatment (88). The response to ERT in FD was proposed as a promising method for monitoring (90), but Gb3 measurement in PBMCs did not represent the best alternative to investigate the Gb3 deposits in Fabry cases with missense mutations (89).

### Globotriaosylsphingosine (LysoGb3)

Analysis of lysoGb3, a degradation product of Gb3, represents an indicator of disease activity as it may reflect the overall total body substrate accumulation (91). Therefore, lysoGb3 is used for diagnostic and screening of Fabry disease, and also in monitoring treated patients, as its level decreases with ERT. It was reported that the measurement of lysoGb3 may be helpful for Fabry diagnosis, particularly in females with normal and/or borderline α-Gal A activity, and that associate non-specific symptoms (92, 93). The study of Stiles et al. showed that plasma lysoGb3 analysis and full gene sequencing are more efficient for the diagnosis of females with Fabry disease compared with α-Gal A enzyme activity (87).

It was suggested that lyso-Gb3 may be used also in the determination of the pathogenicity of a mutation (including heterozygous cases) (91). A recent study performed by Stiles et al. concluded that plasma lysoGb3 is a sensitive and specific biomarker for Fabry disease in males and females, and provides supportive diagnostic information when the results of *GLA* gene sequencing analysis are negative or inconclusive (87). Different studies highlighted the importance of plasma lysoGb3 in elucidating the impact of variants of uncertain clinical significance revealed by molecular investigations and assigning a disease classification (87, 94, 95). Normal lysoGb3 values cannot exclude Fabry disease in female cases but make the diagnosis of Fabry disease in men highly unlikely (96). It was reported that some variants, such as p.S126G, p. D313Y, p.A143T may not result in increased lysoGb3 levels (96). The lysoGb3 level may be measured from DBS by the LC-MS method (91).

### Investigations of Renal Involvement in FD

Albuminuria and/or proteinuria, serum creatinine, glomerular filtration rate, and cystatin C, together with urinary microscopy and renal biopsy are often assessed for diagnostic, evaluation of kidney damage, monitoring of disease progression, and monitoring of treatment (97).

Tubular dysfunction is often underestimated in children with Fabry but it should be analyzed in routine clinical care. All children with Fabry disease should undergo a renal assessment: albuminuria, proteinuria (from 24-h urine collection), and GFR. Other parameters that should be considered are serum urea, creatinine, uric acid, and Cystatin C. For early detection of microalbuminuria, the measurement of the albumin/creatinine ratio in spot urine is recommended. In addition, the creatinine and cystatin C-based GFR-calculation is indicated for the estimation of renal function. Also, abdominal ultrasound should be carried out in these cases. These procedures should be evaluated at the initial clinical workup and follow-up monitoring (62). Considering that early kidney involvement is clinically silent and that early specific therapy is more likely to prevent the progressive damage of the kidney, alternative markers of renal dysfunction are required. Therefore, the research of biomarkers that are correlated with the earliest pathological findings is essential, as these biomarkers can become a non-invasive method of diagnosis in Fabry disease (98, 99).

### Proteinuria

Proteinuria (defined as the urine protein to urine creatinine ratio UPCR) even if it is not a sensitive biomarker for early kidney injury in Fabry nephropathy, is clinically one of the most often used biomarkers of Fabry nephropathy (68, 100). Based on the study of Mehta et al. that included 366 patients with Fabry from 11 European countries, the most frequently reported sign was proteinuria, which was found in 44% of hemizygous males and 33% of heterozygous female patients (101). The degree of proteinuria is an independent risk factor for the extent of declining renal function over time (68, 100). Increased levels of proteinuria before the starting of ERT may be used to predict the renal outcome. As it was reported that cases with more severe proteinuria progress more quickly to ESRD compared to those with mild proteinuria, therefore, treatment of Fabry nephropathy should aim to decrease proteinuria (100, 102). Progression of proteinuria, especially to levels > 1 g/day is a strong predictor of progression to ESRD in Fabry disease (73). It was reported that proteinuria may not be evident in all cases with advanced kidney disease, and not correlate with GFR decline (98). Nephrotic range proteinuria is rare in Fabry patients (100). Proteinuria may start during childhood, even in the first decade of life (60, 103). It was noticed that the responsiveness to ERT may be incomplete after proteinuria is manifest and the level of proteinuria usually does not reach normal values (73, 104). Loss of integrity of the glomerular basement membrane will lead to proteinuria that will have a significant contribution to the progression of renal injury toward tubulointerstitial fibrosis (50).

### Albuminuria

Albuminuria is the most common clinical sign of renal involvement in patients with Fabry disease, often preceding a detectable loss of kidney function, and is related to glomerular and podocyte injuries (6, 97, 100, 105). Albuminuria is not a sensitive biomarker for early kidney damage as it did not discover subclinical phases, as it is detectable only if glomerular podocyte damage is present (6). The kidney biopsy revealed the presence of advanced kidney lesions in Fabry patients despite normal levels of albuminuria (80). Albuminuria is considered to be a more precise indicator of kidney damage in Fabry disease and represents an earlier pathological sign than proteinuria (6, 100). Even if microalbuminuria does not represent an accurate predictor in adults with Fabry, an earlier onset of microalbuminuria in children may have stronger predictive importance (106). Microalbuminuria represents a non-invasive evaluation tool that can be easily performed and that may be an early indicator of kidney disorder and is indicated to be monitored regularly and treated appropriately if present (100).

### Serum Creatinine, Creatinine Clearance, and Glomerular Filtration Rate

Serum creatinine or creatinine clearance may be used to assess renal function. Creatinine clearance is not a confident marker in the evaluation of glomerular filtration rate in children considering the muscle mass changes with age. For estimating the GFR to assess kidney function serum creatinine-based equations for routine clinical use are recommended the Bedside-Schwartz formula for children and The Chronic Kidney Disease Epidemiology Collaboration (CKD-EPI) equation for adults (78, 107). Fabry patients with rapid progression of nephropathy have a higher urinary protein to creatinine ratio (97). The study conducted by Madsen et al. indicated that high urine albumin–creatinine ratio (UACR > 300 mg/g) was associated with a faster reduction of kidney function, in both non-age-standardized and age-standardized analyses, independent of gender (108). Similarly, findings were previously reported by Wanner et al. (109), Germain et al. (110), and Nowak et al. (111).

### Cystatin-C

Cystatin-C, a cysteine small protease inhibitor, comprised of 122 amino acids, is produced by all nucleated cells and is a biomarker of glomerular function related to estimated glomerular filtration rate (eGFR) (86). Cystatin-C has been proposed, as a sensitive and reliable marker of glomerular filtration rate of renal function in Fabry patients receiving ERT and it may be used for the estimation of the efficiency of the ERT (97, 112). It was shown that the level of Cystatin-C does not depend on age, sex, or muscle mass (100, 112). Cystatin-C is not widely used in clinical practice in the management of Fabry nephropathy due to higher costs than those for creatinine dosage and also due to the labor method used for its evaluation, and less available than creatinine (97, 113). In the study of Torralba-Cabeza et al. that included 178 subjects (89 Fabry patients and 89 healthy controls), Cystatin-C concentration was found to be a superior and more sensitive marker than serum creatinine for detection of early renal dysfunction and small decreases in glomerular filtration in both males and female Fabry patients (114).

### Hyperfiltration

Glomerular hyperfiltration may be a common and early sign in young Fabry disease and may represent a marker for Fabry nephropathy (97). The study of Riccio that included 87 Fabry patients revealed that hyperfiltration was correlated with young age, and low proteinuria levels (98). Glomerular hyperfiltration among Fabry patients might help in implementing therapeutic strategies. Different studies observed a decrease in glomerular hyperfiltration after the introduction of ERT, suggesting a positive effect in the long term (115, 116).

### Serum LysoGb3

The level of serum lysoGb3 is increased not only in Fabry disease males but also in many Fabry disease heterozygotes. Serum lysoGb3 represents a biomarker that is easily measured for Fabry disease activity, for monitoring of ERT, and may be useful for treatment evaluation of heterozygotes females (96). Evidence shows that lysoGb3 promotes the proliferation of smooth muscle cells, damages nociceptive neurons, leads to podocyte loss and glomerulus fibrosis, and results in inhibition of endothelial nitric oxide synthase (53). In cases with non-specific Fabry symptoms (for example CKD or LVH) but without characteristic signs or biochemical changes of classical Fabry disease, increased levels of lysoGb3 are suggestive of a diagnosis of Fabry disease. Smid et al. suggested that a normal lysoGb3 cannot exclude Fabry disease in females but makes the diagnosis of Fabry in males highly unlikely (96). The study of Nowak et al. that included 66 genetically confirmed Fabry patients reported that serum lysoGb3 is a significant risk factor associated with important adverse clinical outcomes in a long-term study (68 months), and represents a useful biomarker for diagnosis in heterozygotes with normal α-Gal A enzyme activity (111).

### Kidney Biopsy

Different studies recommend that kidney biopsy should be considered in selected pediatric cases, especially in children with significant proteinuria or a fast decline in renal function, a variant in the *GLA* gene, when the decision to start ERT is doubted and an uncertain diagnosis of Fabry disease (17, 97, 117). Kidney biopsy may be considered in selected pediatric cases, especially in children with significant proteinuria or a fast decline in renal function, when the decision to start ERT is doubted; or when it is necessary to rule out a second renal disorder (17). Kidney biopsy may be considered in patients where the diagnosis can be challenging and in those cases where there is uncertainty about whether to start ERT to identify the Gb3 accumulation (50). In the study of Choi et al. that investigated Fabry disease pediatric patients from South Korea, it was observed that all children presented no proteinuria and normal serum creatinine levels. Kidney biopsy performed in three pediatric male patients before ERT revealed global sclerosis (as seen on light microscopy), while in two cases the accumulation of Gb3 was observed in the mesangial cells on electron microscopy (11). Electron microscopy images may show characteristic/ pathognomonic zebra bodies lamellar deposits (lamellar lipid inclusion bodies) in podocyte cytoplasm and tubules (68).

It is very important to have an early diagnosis of Fabry nephropathy whereas the early initiation of treatment may stop or delay progressive renal dysfunction more effectively compared with the late therapy initiation. Different studies considered that the kidney biopsy with electron microscopy analysis represents the only diagnostic for confirmation or exclusion of Fabry disease nephropathy and recommend to be considered for all patients with CKD, a variant in the *GLA* gene, and an uncertain diagnosis of Fabry disease (97, 117). The study of Thurberg et al. observed after kidney biopsy in children higher storage of Gb3 in distal tubular epithelial cells and podocytes, with the widening of their foot processes (118).

Gb3 deposits may be identified in omiscated epoxy-embedded semithin sections stained with toluidine blue under light microscopy (119). Gb3 deposits emerge as dark blue, dens granules particularly in podocytes, epithelial and tubular cells as well as in smooth muscle and endothelial cells (41). This specific lipid staining examined by common light microscopy is a valuable method when electron microscopy is not available (58).

Light microscopy (periodic acid–Schiff) revealed a wide spectrum of morphological changes in the glomerular, tubulointerstitial, and vascular areas in mild albuminuric children (80). Furthermore, electron microscopy showed a heavy accumulation of Globotriaosylceramide (Gb3) inclusions in podocytes and distal tubular epithelial cells in all patients and lower amounts (slightly increased amounts) in endothelial and mesangial cells (80). Based on these findings, Tøndel et al. (80) endorsed a timely kidney biopsy in all albuminuric patients with FD trying to assess the magnitude of kidney damage (80). Another study documented early renal pathologic lesions by electron microscopy (EM) in young patients with Fabry disease. The same study showed an age-dependent progressive Gb3 inclusions density in podocytes and not in endothelial and mesangial cells (73). Moreover, this study documented the existence of podocytes damage in Fabry patients without albuminuria, therefore this parameter is not sensitive enough for a diagnosis of early Fabry nephropathy (73). In another study, the morphologic changes in the kidney biopsy samples in young patients with Fabry disease treated with agalsidase alfa or agalsidase beta for 5 years were evaluated (119). This study demonstrated a significant correlation between podocyte GL3 clearance and cumulative agalsidase dose in young patients with Fabry disease as well as concomitant reduction of podocyte effacement (119).

Considering that kidney biopsy is invasive and carries some risk for complications it is a controversial subject in children with Fabry disease (6). Recently it was recommended to take into account that kidney biopsy should only be selected in cases where it is under the question to initiate ERT, in those children with marked proteinuria, or those children with a rapid decrease in kidney function or for differential diagnosis (6). Electron microscopy images may show characteristic/ pathognomonic zebra bodies lamellar deposits (lamellar lipid inclusion bodies) in podocyte cytoplasm and tubules (68).

### Urinary Microscopy

Targeted urinary microscopy, a non-invasive, accessible, affordable, and rapid diagnostic test that may be used, in those cases where leucocyte α-Gal A activity and *GLA* genotyping are not available. Different reports suggest that urine microscopy may be useful for the evaluation of the progression of the disorder (120) but its value is limited especially in heterozygous females and due to the fact that the majority of the findings are not specifically characteristic for Fabry disease. Examination of urinary specimens under phase-contrast microscopy using a polarized light filter may provide additional information by identifying birefringent Maltese cross patterns (MC, oval fat bodies) in the urine sediment of patients. Even though it is a valuable method is not a routine evaluation in most clinics evaluating Fabry patients. Selvarajah et al. observed characteristic MC particles inside the vacuolated urinary epithelial cells, with lamellated appearance with protrusions probably due to accumulation of Gb3, namely MC2 particles, and reported high sensitivity and specificity (100%) (120). In addition, Selvarajah et al. suggested that the number of characteristic urinary MC particles increases concomitantly with rising albuminuria, therefore, this investigation may be useful in assessing Fabry's nephropathy progression. The routine laboratory urine assessment by microscopy that does not include a polarized light examination may easily miss the MC particles (120). By using the proper microscopy method also podocyturia may be evaluated (121). Besides microscopy of stained cells, immunohistochemistry, or immunofluorescence methods different techniques may use to assess podocyturia (liquid chromatography coupled with tandem mass spectrometry (LC-MS/MS) method, or mRNA quantification (77, 120, 122, 123).

Elevated levels of podocyturia were observed by Trimarchi et al. in cases with Fabry disease compared to the normal population and suggested that podocyturia may precede proteinuria (77). In addition, Sanchez-Niño et al. showed that podocyturia may precede pathological albuminuria in Fabry disease (124). Fall et all, showed that Fabry disease is associated with increased podocyte loss and reported direct associations between podocyturia and severity of Fabry nephropathy. In addition, in the study of Fall et al. an inverse association was found between podocyturia and eGFR in male Fabry patients (76). It was shown that Fabry cases with ERT had a lower podocyturia, suggesting that ERT may reduce their irreversible loss by stabilizing podocytes' attachment, and suggested that monitoring of podocyturia may be useful for follow-up of Fabry patients on treatment in order to evaluate the treatment response (76, 77).

### Urinary Gb3

Increased levels of urinary Gb3 may be found in Fabry patients with no residual α-Gal A enzyme activity. Normal levels of urinary Gb3 were measured in heterozygous females and those cases with a major residual α-Gal A enzyme activity (3).

Vedder et al. reported that elevated levels of Gb3 in plasma or urine did not correlate with the severity of the disease nor with Fabry-related symptoms. According to the analysis of the Dutch Fabry cohort, it may be considered that urinary or plasma Gb3 levels have no value as surrogate disease markers (3).

The level of urinary Gb3 being reduced after the beginning of enzyme replacement therapy is an indicator of the metabolic effects of treatment. Increased level of urinary Gb3 is associated with the production of α-Gal A antibodies (3, 91, 125).

Simonetta et al. considered that there is no evidence to use urinary Gb3 for the prognostic role or for monitoring the response to enzyme replacement therapy on the nephrological outcome (91). The study of Auray-Blais et al. that included 110 children and adults with Fabry disease found no correlation between the estimated GFR and urinary Gb3 (126). Similarly, changes in urine Gb3 are not useful biomarkers for the prediction of Fabry disease-related changes in eGFR (125). A significant correlation between the levels of urinary excretion of Gb3/creatinine and types of mutations (*p* = 0.0007), sex (*p* < 0.0001), and treatment (*p* = 0.0011) in children and adults with Fabry disease was observed in the study performed by Auray-Blais et al. (126). The levels of urinary lysoGb3 correlate with proteinuria and albuminuria, but not with GFR, therefore it does not represent a good indicator of renal function (126).

### Beta 2-Microglobulin

Beta 2-microglobulin (β2M) represents a biomarker for the tubular reabsorption function (113). Even if it is not usually measured, β2M in serum samples was shown to be the most appropriate renal biomarker for the determination of renal impairment among Fabry disease patients. Considering that β2M is not influenced by muscle mass, it may represent a potential marker that may be used for the estimation of GFR more accurately in adults. Argyropoulos et al. considered that serum β2M as a measure of glomerular filtration function is not useful in children when compared with adults (127).

### Uromodulin

Uromodulin, also known as Tamm-Horsfall protein, is the most abundant protein in human urine, that is exclusively produced in the kidney and its daily secretion is about 50–150 mg (128). An important biomarker for the development of CKD may be represented by the urinary concentration of uromodulin (129). Uromodulin may be a potential urinary biomarker of the renal tubular reserve function in male and female Fabry patients with nephropathy (130).

Doykov et al. reported significantly lower uromodulin levels in Fabry disease cases with both kidney and cardiac involvement (*p* = 0.0045). Lower levels of uromodulin were observed in the late-stage patients presenting kidney, heart, and central nervous system involvement (*p* = 0.0049) (131). In a previous study, uromodulin excretion was reduced in the urine of FD patients (132), while Matafora found a higher urinary excretion of uromodulin in naive Fabry patients (130). Despite these conflicting results, both studies found that urinary excretion of uromodulin came to normal after ERT, with concentrations similar to those observed in healthy controls (130, 132). Based on these the authors (130, 132) concluded/speculated that this urinary protein may be used as a marker to monitor the response to ERT.

Recently, Steubl et al. observed that uromodulin was independently associated with ESRD or rapid loss of eGFR, and suggested that uromodulin might serve as a robust predictor of rapid kidney function decline (129). It was suggested that uromodulin up-regulation could be considered a very early marker of kidney damage at the tubular level, in cases with a normal level of creatinine and normal GFR, especially in heterozygous female Fabry patients, that may associate the normal value of α-Gal A activity (130, 133).

### Prostaglandin H2 D-Isomerase

The excretion of prostaglandin H2 D-isomerase is increased in Fabry disease, probably due to tubular dysfunction present in affected patients (130). The study of Matafora et al. revealed that the prostaGLAndin H2 d-isomerase concentration decreased after ERT speculating that these proteins may be used as markers for monitoring the response to treatment (130).

### Bikunin

Bikunin or urinary trypsin inhibitor (UTI) is a serine protease inhibitor, whose excretion increases in inflammations and accumulates in urine. In pathological conditions such as kidney disease, the concentrations of bikunin in plasma and urine are increased (97). The study of Lepedda et al. that included 24 Fabry cases and 43 healthy controls, showed that urine bikunin levels may be an early biomarker of renal deterioration in Fabry patients (134). The same study performed by Lepedda noticed no association between serum creatinine and urine bikunin levels, therefore, the direct involvement of the kidney in urine bikunin excretion in the case of Fabry's patients is not enough (134).

## Effects of Currently Available Therapies on Renal Symptoms

By its multisystemic character and progressive course, Fabry disease is characterized by a significant reduction in life quality and expectancy. Fabry disease requires a multidisciplinary approach and organ-specific treatment. Specific therapy for Fabry disease should not be initiated in individuals bearing non-pathogenic *GLA* variants.

Currently, available treatment options are represented by enzyme replacement therapy (ERT), and chaperon therapy. Increased efforts were made in the last years for a better therapy in Fabry patients and new treatments that include substrate reduction therapy (SRT) gene therapy, and mRNA based therapy are administrated in clinical trials.

### Enzyme Replacement Therapy

Currently, available enzyme replacement therapies are agalsidase-alpha and agalsidase-beta approved in Europe since 2001. ERT aims to restitute defective α-GAL A (58). Agalsidase alfa (Replagal; Shire Human Genetic Therapies Inc., Lexington, MA, USA/ (Replagal; Takeda Pharmaceutical, Tokyo, Japan), with a certified dose of 0.2 mg/kg, in male pediatric patients over the age of 7, or agalsidase beta (Fabrazyme; Genzyme, a Sanofi company, St Germain en Laye, France) at the licensed dose at 1.0 mg/kg in pediatric patients ≥ 8 years of age. ERT is recommended to be administered every other week in a short intravenous infusion.

According to US consensus, the ERT should be considered in all symptomatic cases regardless of age or gender (17). In asymptomatic boys with Fabry disease with *GLA* mutation, based on US consensus, the ERT should be recommended around the age of 8–10 years (17). According to the European Fabry Working Group consensus in asymptomatic boys with classic mutation for Fabry, the ERT treatment should be started at the age of 16 (135).

The initiation of ERT therapy as early as possible gives the best clinical outcome and ERT's effect depends on the stage of the disease (85). Prevention and early specific therapy, ERT, are important, as they may slow progressive symptomatic organ complications, improve quality of life and in later life, improve both morbidity and mortality, and preserve life expectancy. ERT should be considered in the case of symptomatic boys and girls that present neuropathic pain, pathological albuminuria (≥3 mg/mmol creatinine), severe gastrointestinal symptoms, and abdominal pain or cardiac involvement. Initiation of ERT based only on the presence of angiokeratoma is not recommended in children (62).

Different studies with agalsidase-alfa showed decreased Gb3 accumulation in the liver and in tubular epithelial cells and a reduction of Gb3 excretion in urine, a notable reduction in podocytes Gb3 inclusions, and complete clearance of glomerular endothelial and mesangial inclusions (49, 119, 136).

It was shown that ERT reduced Gb3 in the kidneys, heart, and skin, being particularly effective in clearing the endothelial cells. On the other hand, podocytes, distal tubular cells, and smooth muscle cells showed a smaller reduction of Gb3 than that observed in other cell types therefore it seems to be more resistant to ERT (118, 136).

The clinical benefit of ERT is mainly observed in patients who start ERT before the presence of irreversible organ damage (137). Arends et al. observed that despite treatment with ERT disease progression is predicted by the presence of reduced renal function, and proteinuria at the time of therapy initiation (138).

Unfortunately, in response to ERT, immunoglobulin G antibodies may be generated (in about 40% of Fabry males with no α-Gal A activity) and lead to inhibition of enzyme activity that may negatively influence the clinical outcome of Fabry patients (139).

In the non-pathogenic *GLA* variant carriers (for example p.E66Q, p.R118C, p.S126G, p.A143T, and p.D313Y) ERT should not be started (62). Two new forms of ERT, with increased stability and lower immunogenicity, for the treatment of Fabry disease have been developed; Pegunigalsidase-alfa (PRX-102, Protalix Biotherapeutics, Israel and Chiesi Global Rare Diseases, USA) and moss-agalactosidase A (moss-aGal, Greenovation biopharmaceuticals, Germany). It was suggested that PRX-102 may stabilize renal function and that moss-aGal may target kidney cells (140, 141). Despite ERT treatments (agalsidase alfa and beta) that are associated with anti-drug antibody (ADA) development that is associated with reduced pharmacodynamic and clinical responses, the new ERT pegunigalsidase-alfa is associated with low immunogenicity, improvements in symptoms, and also with an important reduction in Gb3 deposition in the kidney and a reduction in plasma lysoGb3 level (140).

### Chaperone Therapy

Chaperone therapy with Migalastat (Galafold, Amicus Therapeutics), was approved in 2016 in Europe and 2018 in the USA, respectively. In European Union, Migalastat was approved first for Fabry patients with an amenable mutation, older than 16 years. In 2021 Migalastat's (Galafold) approval was expanded to FD children with an amenable mutation, starting at age 12 and weighing at least 45 kilograms (99 pounds). Chaperone therapy may be used in patients with “amenable” variants (for example p. N215S) that are missense mutations with normal α-Gal A catalytic activity, but these mutations lead to a reduction in overall α-Gal A enzymatic activity due to strongly decreased stability of the mutated protein (142) Based on the review of Weidemann, it is important to classify the *GAL* gene mutation for amenability to treatment with Migalastat in each new Fabry case (142). Non-amenable *GAL* gene mutations (for example (large mutations, frameshift mutations, splicing mutations, insertions, truncations) do not pass the good laboratory practice (GLP) HEK assay for amenability developed by Benjamin et al. (143).

A study by Riccio et al. showed a significant decrease in proteinuria after 1-year of therapy with Migalastat (144). Migalastat is significantly eliminated by the kidneys and is not recommended in Fabry cases with GFR <30 ml/min/1.73 m^2^ or ESRD that requires dialysis. In the case of GFR >30 ml/min/1.73 m^2^ is not necessary the dose adjustment (41). Migalastat is the only oral treatment for Fabry, that may be used as first-line treatment in ERT-naive patients, or as an alternative to ERT (144). Migalastat represents an alternative to ERT in cases where ERT response is lost or in cases of antibody formation to ERT (42). Migalastat stabilizes α-GalA mutated protein, escaping from degradation and movement to the lysosomes (136). Migalastat as monotherapy has been shown to reduce the Gb3 accumulation but for achieving its effects, the drug requires at least some endogenous enzyme production. Thus, only a limited group of Fabry patients would respond to monotherapy with Migalastat. Migalastat is recommended for selected patients, without mutations that cause complex enzyme alterations (145). In the future, a possible approach for increasing the stability of the α-Gal A enzyme activity may be represented by the co-administration of Migalastat and ERT. The study of Warnock et al. revealed an increase of α-Gal A plasma level of 1.2–5.1 fold in cases that received combined Migalastat and ERT compared with those that received only ERT (146). The study of Benjamin et al. indicated that the co-administration of ERT and migalastat in Fabry mice resulted in an increased AGAL tissue uptake and improved Gb3 reduction (147).

Chaperones favor the proper folding of the mutated protein and increase its stability, which results in a reduction of Gb3 and its substrates. Fabry patients which are eligible for treatment with Migalastat are established by using an *in vitro* enzyme activity assay (122). Chaperone Migalastat is an inhibitor of α-GAL A, but in small doses, it may increase α-GAL A enzymatic activity for some *GLA* gene mutations (142).

### Substrate Reduction Therapy

Additional therapy is represented by substrate reduction therapy (SRT) which targets the glycosphingolipid synthesis to reduce the formation of metabolites that cannot be degraded. SRT is only available in clinical studies (139, 140) and it is not licensed to treat patients at the moment. In patients with residual enzyme activity, SRT may be enough as a single therapy for reducing the level of the substrate. SRT drugs that are used in clinical trials are Lucerastat and Venglustat.

Lucerastat or N-butyldeoxygalactonojirimycin (Idorsia Pharmaceutical Ltd, Allschwil, Switzerland) functions as an inhibitor of glucosylceramide synthase and thus will prevent the accumulation of Gb3 and lysoGb3 by restricting the quantity of ceramide that is metabolized in glycosphingolipid and is currently under evaluation in phase three clinical study for Fabry disease (139). Recently it was reported the reduction of sphingolipids and their metabolites after initiation of SRT at doses of 1,000 mg twice a day as single therapy (136). It was suggested that in addition to ERT may provide a new form of combination therapy that could be beneficial to this population.

Venglustat (Ibiglustat, Sanofi Genzyme, Cambridge, MA), an oral inhibitor of glucosylceramide synthase prevents the synthesis of glucosylceramide (GL1) and therefore reduces Gb3 (139) and is under investigation in phase two clinical study.

In the case of patients with Fabry disease with specific (“amenable”) mutations, treatment with chaperones may represent the appropriate approach while for the remaining Fabry patients, the combined therapy such as ERT with substrate reduction therapy, might have a benefic effect.

### Gene Therapy

Gene therapy, a promising option for treatment, aims to correct the underlying genetic defect of Fabry disease. Theoretically, gene therapy could be more effective than current options in Fabry disease due to the fact that transduced cell populations will produce α-Gal A continuously. A pilot study (NCT02800070) that included five patients with type I Fabry disease used lentiviral vectors for carrying the coding sequence for α-Gal A. The patients received autologous transduced CD34^+^-selected cells that were genetically engineered to express α-Gal A and the transduced cells may provide the functional α-Gal A enzyme to sites that are not accessible to ERT therapy. The trial showed that the Fabry patients' recipients had plasma and leukocyte normal α-Gal A activity and associated reductions of plasma and urine Gb3 and lysoGb3 levels (148). Due to the previous studies that revealed the persistence of adeno-associated viruses for multiple years and considering that they lead to therapeutic levels of transgene expression in 2019 two clinical trials were approved for the treatment of Fabry disease. Those clinical trials utilized adeno-associated viruses-based gene therapy or adenovirus encoding *GLA* cDNA are administrated for cellular expression of αGal A in different organs such as kidneys, lives, heart, etc (149). Therefore, gene therapy may represent in the future an effective treatment option for patients with Fabry disease. Gene therapy is associated with some risks, the most significant are toxicity and immune reactions. Virus vectors used may induce inflammatory responses, stimulating the production of antibodies that may lead to a decreasing efficacy. The integration of the functional gene into the host genome may disrupt the host gene at the site of insertion or may activate o proto-oncogene and promote malignancy development (6, 150).

### mRNA Therapy

mRNA therapy, with drug-like properties, induces a prolonged human α-galactosidase protein production and subsequent secretion. Multi-component lipid nanoparticles were developed for the distribution of mRNA that encodes a human α-galactosidase protein (149). It was observed that administration of lipid nanoparticles mRNA for human α-galactosidase increased α-Gal A levels expressed in different tissues (kidney liver, heart) and led to an improved Gb3 clearance (151). Unfortunately, mRNA-based therapy has a transient effect therefore repeated administration is required. In the future, mRNA therapy may represent an alternative approach to the treatment of Fabry disease.

### Supportive Therapy

Simultaneously with specific therapy, supportive care should be offered to manage pain, gastrointestinal symptoms, arterial blood pressure, and, when kidney failure occurs, renal replacement therapy and transplantation (Table 5).

**Table 5:** Symptomatic therapy in pediatric patients with FD.

**Aim/ target**	**Therapy**	**Monitoring/** **Assessment/** **Evaluation**	**Reference**
Underlying disorder	ERT Migalastat		
Neuropathic pain (acroparesthesia, pain, etc)	Acetaminophen, NSAIDs ERT, anti-epileptics, tricyclic antidepressants, serotonin-norepinephrine uptake inhibitors, carbamazepine	Neurological exam	(62, 152)
Kidney-proteinuria	ACEI, ARB, ERT	UACR, UPCR,	(41, 62)
Kidney-ESRD	RRT (dialysis, transplantation)	Urea, creatinine, Cystatin C, eGFR, US	(62)
Arterial hypertension	ACEI, ARB,	BP monitoring, ABPM	(41)
Dysrhythmia	Antiarrhythmic drugs Bradycardia-pacemaker implantation	ECG, Holter test	
GI symptoms (abdominal pain and diarrhea)	ERT, dietary restrictions, small meals; metoclopramide, H2 blocker	Clinical exam	(62)

*ACEI, angiotensin enzyme converting inhibitors; ARB, angiotensin receptor blocker, RRT, renal replacement therapy, NSAID , nonsteroidal anti-inflammatory drugs; BP, blood pressure; UACR, urinary albumin to creatinine ratio; UPCR, urinary protein to creatinine ratio, ECG, electrocardiogram; ABPM, ambulatory blood pressure monitoring*.

A low sodium diet is recommended in Fabry patients considering that sodium may reduce the effects of ACEI, and ARB and may predispose/increase the risk for ERDS in cases with proteinuria (139). Blood pressure should be monitored when starting ACEI or ARB treatment, and it is recommended to avoid the combination of both drugs as they may lead to additive drug-related adverse effects The ACEI or ARB administration should be given at bedtime in Fabry patients with low blood pressure (41). It is recommended for Fabry patients to avoid the use of nephrotoxic substances, non-steroidal anti-inflammatory drugs (NSAID) such as ibuprofen that may lead to acute kidney injury. Because of avoidance of sunlight exposure and malabsorptive gastrointestinal disease in Fabry patients, vitamin D deficiency should be checked and vitamin D supplementation should be provided if it is necessary. Vitamin D receptor activation reduces the inflammation associated with lysoGb3 and, therefore, Fabry patients should be screened for and started on the appropriate supplementation regimen if needed (153).

## Monitoring

It is recommended to monitor boys at least once a year and girls at least every 2–3 years. In the case of asymptomatic children from families with members diagnosed with Fabry disease, Germain et al. recommend that the baseline assessment of organ involvement should begin at 5 years of age for boys and at 12–15 years of age for girls (62).

## Conclusion

FD is a multisystemic and multifaceted disease that starts early in life, with symptoms occurring during childhood with progressive evolution that worsens throughout adulthood. Nowadays with early diagnosis of kidney involvement in FD and new proposed therapies a better outcome is expected.

## Author Contributions

CM and CB: ideas, coordination of the study, writing and editing the manuscript, analysis of the data, and writing up the results. CM, IS, and CS: coordination of the study between three centers and writing the manuscript. CM, IS, CS, and CB: write and revised the manuscript. All authors have read and agreed with the final form of the manuscript.

## Funding

This was partly supported by a project financed by the Romanian Ministry of Education and Research, CNCS—UEFISCDI, project no PN-III-P4-ID-PCE-2020-1928, within the PNCDI III, contract no. PCE 72/2021.

## Conflict of Interest

The authors declare that the research was conducted in the absence of any commercial or financial relationships that could be construed as a potential conflict of interest.

## Publisher's Note

All claims expressed in this article are solely those of the authors and do not necessarily represent those of their affiliated organizations, or those of the publisher, the editors and the reviewers. Any product that may be evaluated in this article, or claim that may be made by its manufacturer, is not guaranteed or endorsed by the publisher.

## Footnotes

^1^Available online at: https://www.ncbi.nlm.nih.gov/clinvar?term=300644[MIM].
