# Corrigendum: Clinical characteristics, renal involvement, and therapeutic options of pediatric patients with Fabry disease

**DOI:** 10.3389/fped.2022.1045199

**Published:** 2022-10-19

**Authors:** Carmen Muntean, Iuliana Magdalena Starcea, Cristina Stoica, Claudia Banescu

**Affiliations:** ^1^ Department of Pediatrics I, George Emil Palade University of Medicine, Pharmacy, Science, and Technology of Targu Mures, Targu Mures, Romania; ^2^ Department of Pediatric Nephrology, Sf Maria Emergency Hospital for Children Iasi, University of Medicine and Pharmacy Grigore T. Popa Iasi, Iasi, Romania; ^3^ Pediatric Nephrology Department, Fundeni Clinical Institute, University of Medicine and Pharmacy Carol Davila Bucharest, Bucharest, Romania; ^4^ Center for Advanced Medical and Pharmaceutical Research, George Emil Palade University of Medicine, Pharmacy, Science and Technology of Targu Mures, Targu Mures, Romania

**Keywords:** Fabry, kidney, GLA gene, biomarker, therapy

A corrigendum on Clinical characteristics, renal involvement, and therapeutic options of pediatric patients with Fabry disease By Muntean C, Starcea IM, Stoica C, Banescu C. (2022) Front Pediatr. 10: 908657. doi: 10.3389/fped.2022.908657

In the published article, authors mistakenly omitted the **Funding** statement. The **Funding** statement appears below.

**Funding**

“This was partly supported by a project financed by the Romanian Ministry of Education and Research, CNCS—UEFISCDI, project no PN-III-P4-ID-PCE-2020-1928, within the PNCDI III, contract no. PCE 72/2021”.

The authors apologize for this error and state that this does not change the scientific conclusions of the article in any way. The original article has been updated.

